# Metal chalcogenides (CuS or MoS_2_)-modified TiO_2_ as highly efficient bifunctional photocatalyst nanocomposites for green H_2_ generation and dye degradation

**DOI:** 10.1038/s41598-023-34743-2

**Published:** 2023-05-17

**Authors:** Reem A. El-Gendy, Haitham M. El-Bery, Mostafa Farrag, Dina M. Fouad

**Affiliations:** 1 Advanced Multifunctional Materials Laboratory, Chemistry Department, Faculty of Science, Assiut University, Assiut, 71515 Egypt; 2 Basics Science Department, School of Biotechnology, Badr University in Assiut, Assiut, 2014101 Egypt; 3 Department of Chemistry, Faculty of Science, Assiut University, Assiut, 71516 Egypt

**Keywords:** Chemistry, Energy science and technology, Materials science

## Abstract

Herein, we report the modification of TiO_2_ nanostructures with two different metal chalcogenides (CuS or MoS_2_). The effect of the preparation scheme (hydrothermal and coprecipitation methods) and the mass ratio of metal chalcogenides were investigated. The as-synthesized photocatalyst nanocomposites were fully characterized by various techniques. Moreover, the photo/electrochemical analysis were performed to investigate the photoelectric properties and photocatalytic mechanism. The photocatalytic performance was evaluated using two test reactions. In the case of H_2_ generation via water splitting, it was found that 0.5 wt% CuS-TiO_2_ synthesized via the coprecipitation method exhibited an initial hydrogen evolution rate (HER) of 2.95 mmol h^−1^ g^−1^. While, the optimized 3 wt% MoS_2_-TiO_2_ synthesized by the hydrothermal method, showed an HER of 1.7 mmol h^−1^ g^−1^. Moreover, the degradation efficiency of methylene blue dye was 98% under UV–Vis light irradiation within 2 h over 0.5 CT_PP and 3MT_HT. Under visible irradiation, the degradation efficiency was 100% and 96% for 3MT_PP and 0.5CT_HT in the presence of H_2_O_2_, respectively. This study has proven that metal chalcogenides can act as effective, stable, and low-cost bifunctional co-catalysts to enhance the overall photocatalytic performance.

## Introduction

The accessibility of clean water and renewable energy sources are considered two of the major challenges facing humanity in the twenty-first century^1^. Nowadays, the development of industry depends strongly on fossil fuels. However, the fossil fuel sources may be depleted in the future. Moreover, the increasing utilization causes extensive greenhouse gas emissions. The development of eco-friendly and environmentally source of energy is highly desirable. Recently, Semiconductor-based photocatalysis has been investigated a promising strategy for water purification and hydrogen production^2,3^.

Since the pioneering work by Honda and Fujishima in 1972 in the splitting of water over n-type TiO_2_ electrodes^4^, several studies have been carried out to explore suitable materials. Many semiconductors, TiO_2_, CdS, and ZnO, have been developed for hydrogen generation and water treatment^5^.

TiO_2_ is considered as the most extensively used photocatalyst because of its availability, great chemical stability, and non-toxicity^6–8^. However, TiO_2_ suffers from an insufficient response to visible light because it has a wide band gap (*E*_0_ = 3.2 eV), the high recombination rate of photoexcited charge carriers, and the rapid reverse reaction. Thus, several attempts have been made to enhance the photocatalytic activity of TiO_2_^9–12^. Due to these limitations, several schemes have been made to alter TiO_2_ with noble metals (e.g., Pt, Pd, Au, and Ag)^13,14^, transition metals (e.g., Cu and Ni)^15,16^ and their oxides (e.g., NiO and CoO_x_)^17,18^, dye sensitization^19^ and the construction of heterojunctions with other semiconductors^20,21^. Many studies employed noble metals and their oxides (e.g., Pt, Pd, RuO_2_, and Ag_2_O)^14,22–24^ as potential co-catalysts to elevate the photocatalytic activity of TiO_2_. However, noble metals are very expensive; thus, modification with non-noble metals as a co-catalyst is urgent.

In recent years, metal chalcogenides photocatalysts, such as CdS, CuS, MoS_2_, and ZnS, have attracted a considerable attention due to their efficient photocatalytic activity toward water splitting and wastewater treatment. These properties can be explained owing to their narrow band gaps, suitable physical and chemical structure, sufficient thermal stability and good response to visible light^25–28^. Therefore, the coupling of metal sulfides with TiO_2_ has played a crucial role encouraging the separation of photogenerated electron–hole pairs and elevating the photocatalytic activity for dye degradation and hydrogen generation. Among these metal sulfides, CuS is a metal sulfide semiconductor with a narrow band gap (2.0–2.2 eV), which is nontoxic, cheap and available^29^. Moreover, CuS/TiO_2_ form type II heterojunction as a result the separation of the photoexcited carriers has been improved. For MoS_2_, it is a 2D layered structure semiconductor with an indirect band gap of 1.2 eV and a direct band gap in the mono-layered form of 1.8 eV. Hu et al. reported that MoS_2_ nanosheets dramatically enhance the performance of CdS to 49.8 mmol h^−1^ g^−1^ for photocatalytic H_2_ evolution^30^. Furthermore, MoS_2_ has also been investigated to be coupled with TiO_2_ to enhance the photocatalytic hydrogen generation and dye degradation under the irradiation of both ultraviolet and visible light^31,32^. Although, many reports have studied the improved photocatalytic activity of TiO_2_ modified with metal sulfides, these studies focused mainly on synthesis methods involving long preparation times and high-temperature conditions. The facile coprecipitation method is a simple technique to prepare TiO_2_ loaded by CuS and MoS_2_ co-catalysts with high activity toward H_2_ generation and dye degradation.

Herein, we demonstrate the modification of TiO_2_ using metal sulfides (MS_x_) co-catalysts (MoS_2_ and CuS) via two different preparation methods: hydrothermal method (HT) and coprecipitation method (PP) with varying the weight ratios of MS to TiO_2_. The H_2_ generation via water splitting was performed under the irradiation of UV light and methanol as a scavenger reagent. Meanwhile the photocatalytic degradation of methylene blue (MB) aqueous solution was performed under both UV–Vis and only Vis irradiation. Also the Photoelectrochemical measurements PEC have been studied. The characterization and the probable reaction mechanism of the as-prepared photocatalysts are discussed in detail.

## Experimental section

### Materials

TiO_2_ Aeroxide p25 (ACROS ORGANICS), Cu(NO_3_)_2_⋅3H_2_O (> 95%), (NH_4_)_6_Mo_7_O_24_⋅4H_2_O (<95%) were obtained from Fisher chemicals, Na_2_S (99%, ALPHA CHEMICALS), C_2_H_5_NS (98%, Alfa Aesar), H_2_O_2_, methanol, and MB was obtained from Sigma–Aldrich. All the reagents were of analytical grade and used as received without further purification.

For comparison purposes metal chalcogenides MS_x_ (CuS or MoS_2_) were loaded over TiO_2_ via two different methods, namely, hydrothermal treatment and coprecipitation methods. The detailed synthesis procedure is illustrated as follows.

### Preparation of MS_x_ (CuS or MoS_2_)/TiO_2_ by hydrothermal method

The MS_x_/TiO_2_ photocatalysts were synthesized through a simple hydrothermal treatment procedure^33^. Typically, TiO_2_ p25 powder was dispersed in 70 mL deionized (DI) water and ultrasonically treated for 10 min, then various quantity ratios of Cu(NO_3_)_2_⋅3H_2_O or (NH_4_)_6_Mo_7_O_24_⋅4H_2_O and an excess of C_2_H_5_NS (Cu/S, Mo/S = 1:2, 1:5 molar ratio, respectively) were dropped slowly onto the above solution. The mixed solution was stirred for 1 h. Subsequently, the mixture was poured into a Teflon-lined autoclave and heated at 160 °C for 12 h (CuS/TiO_2_) and 180 °C for 24 h (MoS_2_/TiO_2_). The resulting product was separated by centrifugation, washed several times with ethanol and water, and dried overnight at 80 °C in air. The resulting photocatalysts were labeled xCT_Z, and yMT_Z, where x = (0.3%, 0.5%, 0.7% and 1.0%); y = (1.0–4.0%) are the weight percent of the hetero-photocatalyst and CT represents CuS/TiO_2_, MT means MoS_2_/TiO_2_, while Z is the preparation method: HT for hydrothermal treatment method and PP for the coprecipitation method.

### Preparation of MS_x_ (CuS, MoS_2_)/TiO_2_ by coprecipitation method

In a typical procedure, TiO_2_ p25 was dispersed in 100 mL (DI) water and sonicated for 10 min, and then Cu(NO_3_)_2_⋅3H_2_O or (NH_4_)_6_Mo_7_O_24_⋅4H_2_O were added and stirred for 30 min after that, a Na_2_S solution was added to the overhead mixture and stirred vigorously for 1 h. The precipitate was washed by centrifugation with ethanol and distilled water, finally dried at 80 °C overnight. In the case of MoS_2_/TiO_2_ 1 ml of HCl (1 M) was added to ease the precipitate formation.

### Photocatalytic hydrogen evolutions

The H_2_ evolution experiments were accomplished in a 200 ml Pyrex reactor. A UV-LED lamp (25 W, 365 nm, NICHIA, Japan) was used as the light source, which was located 1 cm away from the reactor. Typically, 50 mg of the catalysts were suspended in 200 ml of a 20 vol% aqueous solution of methanol as a sacrificial reagent under sonication. The suspension of photocatalysts was initially purged with argon gas (99.99%) for 30 min (100 mL min^−1^) to ensure the removal of all oxygen under magnetic stirring. This photocatalytic reaction was performed for 5 h. The amount of H_2_ produced was measured by gas chromatography (GC-2014, Shimadzu, Japan, with a TCD detector, argon as a carrier gas) every 15 min. The apparent quantum yield (AQY) was measured under similar photocatalytic reaction conditions under various light sources with wavelength of 365, 400, 450, 500, and 520 nm and estimated using the following formula:

$$\mathrm{AQY }\left(\mathrm{\%}\right)= \frac{2\times \mathrm{the \; number \; of \; evolved } \; {\mathrm{H}}_{2} \; \mathrm{ molecules}}{\mathrm{the  \;number \; of \; incident \; photons}}\times 100$$

### Photocatalytic degradation of MB

The as-prepared photocatalysts were used for the photocatalytic degradation of MB under UV–Vis light irradiation. The light source was a 450 W medium-pressure mercury lamp. The distance between the reaction cell and the light source was approximately 10 cm. First, 50 mg of the photocatalyst was injected into 50 mL of 50 ppm MB aqueous solution. Initially, the suspension was treated ultrasonically for 30 min and the mixture was stirred magnetically in the dark for 30 min to reach adsorption/desorption equilibrium. The suspension was exposed continuously to light illumination until complete decolorization of the dye solution. At an appropriate interval 4 mL of the suspensions were withdrawn and centrifuged to remove the photocatalyst powder. The concentration of MB was measured using UV–Vis absorption spectra (Lambda-40, Perkin Elmer, USA). The photodegradation of MB dye was also studied under only visible light illumination using the same lamp with a ˂ 420 nm UV cut-off filter (0.72 M NaNO_2_ solution) in the presence of 5 ml H_2_O_2_. The degradation efficiency of the as-prepared photocatalysts was calculated using the equation:

$$ {\text{Removal }}\left( \% \right) \, = \, ({\text{C}}_{{\text{o}}} - {\text{ C}}_{{\text{t}}} )/{\text{C}}_{{\text{o}}} \times {1}00 $$

where, C_ο_ and C_t_ are the initial concentration of MB and the MB concentration at a specific time, respectively^34^.

### Photoelectrochemical measurements PEC

Photoelectrochemical experiments were conducted using a three-electrode configuration in which platinum wire, saturated Ag/AgCl electrode, and as-synthesized photocatalysts deposited on a fluorine-doped tin oxide (FTO) conducting glass substrate served as the counter, reference, and working electrodes, respectively. In addition, the used electrolyte was 0.1 M Na_2_SO_4_ aqueous solution. The light irradiation source equipped was UV-LED lamp (25 W, 365 nm, NVMUR020A, NICHIA, Japan). Prior to the measurement, the electrolyte was bubbled with argon gas to eliminate all dissolved air. To prepare the working electrode, 20 mg of the as-synthesized catalyst was ultra-sonicated with 1 ml of isopropanol to obtain slurry. Subsequently, a 200 μl of the slurry (four layers, 50 μl for each layer) was dropped onto a fixed area of FTO glass 1 cm^2^, and then dried at 100 °C for 1 h.

Electrochemical impedance spectroscopy (EIS), Mott–Schottky (MS) analysis, Chronoamperometry (CAM), and cyclic voltammetry (CV) were measured using a potentiostat workstation (CorrTest Instruments, model CS350) and CS studio software. The EIS spectra were obtained over a frequency range from 100 kHz to 10 mHz. The photocurrent response was implemented at a potential of + 0.6 V vs. Ag/AgCl.

### Instrumentation and characterization

The crystallographic phase of the as-synthesized photocatalysts was detected with Philips 1700 version diffractometer (40 kV and 30 mA using Cu Ka radiation), the XRD pattern was acquired in the 2θ range of 4° to 80°. The UV–Vis diffuse reflectance spectra (DRS) were conducted on Evolution 220 spectrophotometer (Thermo Fisher Scientific, UK) in the range of 200–1100 nm. The specific surface areas and porosity of the samples were obtained by N_2_ adsorption–desorption isotherm at 77 K using the Brunauer–Emmett–Teller (BET) and Barrett–Joyner–Halenda (BJH) methods (Quantachrome Instrument Corporation, Nova 3200, USA instrument). Prior to surface area analysis, the powders were degassed at 150 °C for 2 h. The morphological characteristics of the as-prepared samples were investigated using transmission electron microscopy (JEOL JEM-2100F). The electrons beam was accelerated to typically 200 kV. The photodegradation rate of MB solution was followed using Lambda-40 UV–Vis spectrophotometer (Perkin Elmer, USA). The chemical composition of the samples was carried out using X-ray photoelectron spectroscopy (XPS, Thermo Scientific, USA, Kα with monochromatic X-ray (Al Kα radiation of − 10 to 1350 eV) with spot size 400 µm, pressure 10^−9^ bar, full-spectrum pass energy 200 eV, and narrow-spectrum 50 eV. The photoluminescence spectra (PL) were probed using Cary Eclipse, Agilent USA fluorescence spectrophotometer.

## Results and discussion

### Photocatalysts characterization

**Figure 1:**
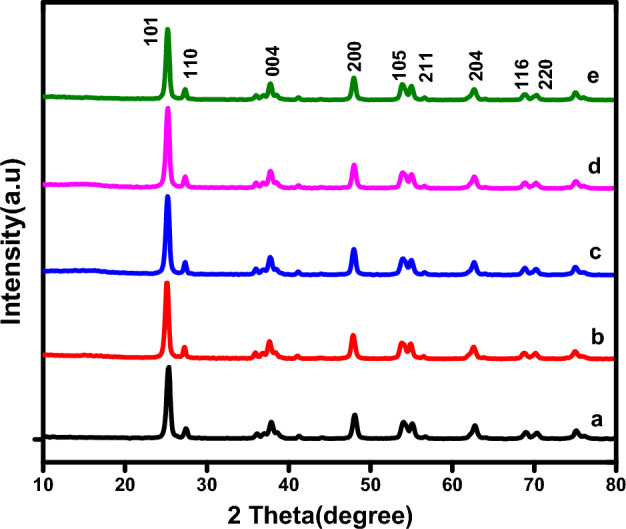
XRD patterns of the as-prepared photocatalysts: (**a**) TiO_2_, (**b**) 0.5CT_PP, (**c**) 0.5CT_HT, (**d**) 3MT_HT, and (**e**) 3MT_PP nanocomposites.

The crystal structure and composition of the as-prepared samples were analyzed using X-ray diffraction technique. Figure 1 displays the XRD patterns of the pure TiO_2_ P25, CuS/TiO_2_, and MoS_2_/TiO_2_ photocatalysts. The diffraction peaks were indexed to TiO_2_ p25; the characteristic peaks were located at approximately 25.3°, 37.9°, 48.04°, 54.1°, 55.1°, 62.72°, and 68.99° 2θ were indexed to the (101), (004), (200), (105), (211), (204), and (116) planes of anatase TiO_2_ (JCPDS No. 71-1167), respectively. The peak at 27.34° 2θ (110) was assigned to rutile TiO_2_^35^. No distinct phase change in TiO_2_ was observed. In addition, the as-prepared photocatalysts exhibited negligible diffraction peaks for MoS_2_ and CuS, owing to the low loading and the high dispersion of CuS and MoS_2_ nanoparticles on the surface of TiO_2_^26^. The XRD patterns of CuS and MoS_2_ are shown in Fig. S1.

**Figure 2:**
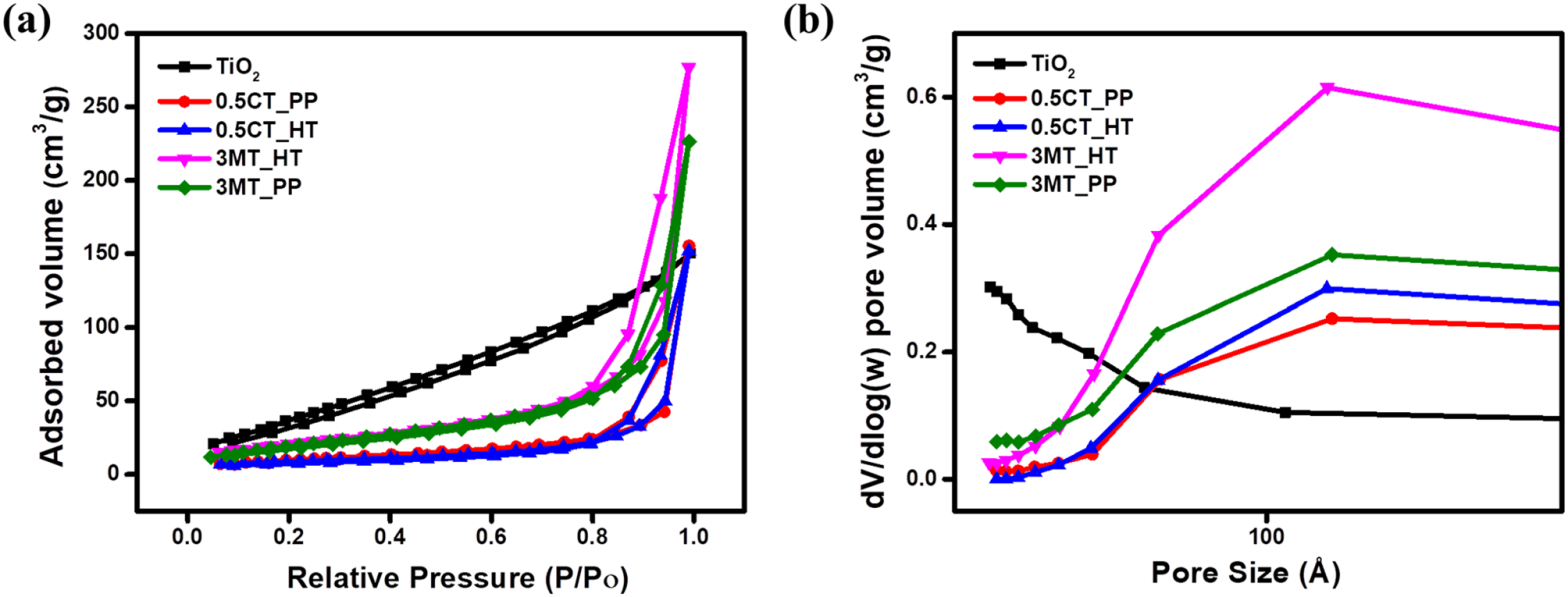
(**a**) N_2_ adsorption/desorption isotherm and (**b**) BJH pore size distribution curves of the as prepared photocatalysts.

N_2_ adsorption–desorption studies were carried out to determine the specific surface area (BET) and the average pore size distribution profiles of the modified photocatalysts. Figure 2a suggests that the curves of the prepared catalysts were attributed to type IV isotherm with a H3 hysteresis loop and relative pressure (*P*/*P*_o_) in the range of 0.7–1.0, indicating a mesoporous structure (2–50 nm)^36^. TiO_2_ had the highest BET specific surface area (162.1 m^2^ g^−1^). The surface area decreased significantly compared to the as-prepared photocatalyst composites: 36.3, 30, 75.34, and 77.4 m^2^ g^−1^ for 0.5CT_PP, 0.5CT_HT, 3MT_PP, and 3MT_HT, respectively, as listed in Table 1. This decrement in BET specific surface area may be attributed to the distribution of CuS or MoS_2_ particles inside the pores of p25 leading to coverage of external surface area, as reported elsewhere^31^.

**Table 1:** Summaries of texture properties and optical band gap.

Samples	S_BET_ (m^2^/g)	Pore volume (cm^3^/g)	Pore size (nm)	Band gap (eV)
P25	162.12	0.21	1.58	2.92
0.5CT_PP	36.30	0.23	6.75	2.83
0.5CT_HT	29.99	0.23	11.83	2.22
3MT_PP	75.34	0.34	6.68	1.82
3MT_HT	77.40	0.42	6.71	2.55

The adsorption–desorption isotherm of different percent’s of CT_PP and MT_HT nanocomposites are shown in Fig. S2. Moreover, the pore size distribution curves of the as-prepared photocatalysts were measured using BJH method (Fig. 2b). It was found that the pore radius of the as-prepared samples increased to a wide range compared to TiO_2_ (Table 1). This was due to the coexistence of CuS or MoS_2._ Moreover, the total pore volume also increases due to the creation of additional pores that enhance the photocatalytic activity. Table 1 lists the corresponding BET specific surface area, total pore volume, and pore radius.

**Figure 3:**
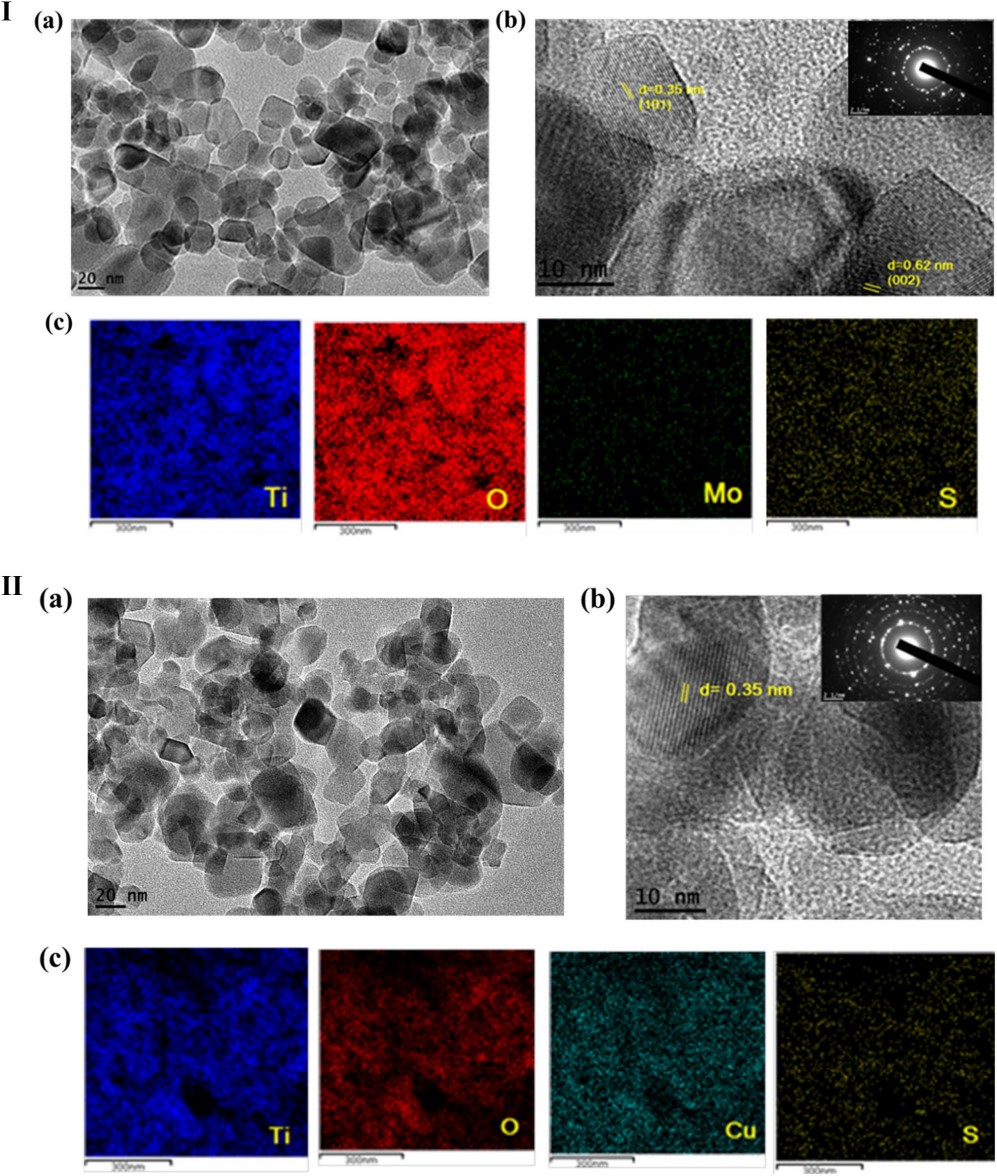
(**Ι**)—TEM (a), HRTEM image (b) and EDX spectrum (c) of 3MT_HT, respectively. (**ΙΙ**)—TEM (a), HRTEM image (b) and EDX spectrum (c) of 0.5CT_PP, respectively.

The morphology and particle size of the as-synthesized CuS/TiO_2_ and MoS_2_/TiO_2_ were measured by TEM, HRTEM and energy-dispersive X-ray spectrometry (EDX) analysis. Figure 3.Ι displays the TEM image of the 3MT_HT nanocomposite synthesized by the hydrothermal method. Well-distributed dark particles of MoS_2_ were observed on the surface of TiO_2_ (Fig. 3.Ι.a). Moreover, the HRTEM image (Fig. 3.Ι.b) displays the lattice fringe spacing of TiO_2_ (0.35 nm) that was attributed to the (101) plane of anatase TiO_2_ (JCPDS No. 71-1167)^37^. Whereas, the lattice fringe distance of MoS_2_ was 0.62 nm corresponding to (002)^38^. The selected area electron diffraction (SAED) suggests the nanocrystalline nature of the modified photocatalyst. Furthermore, the chemical composition of 3MT_HT heterostructure has been investigated by EDX, which revealed the presence of Ti, O, Mo, and S. On the other hand, the crystal phase corresponding to CuS is hard to be observed in the TEM image of 0.5 CT_PP (Fig. 3.ΙΙ.a,b). This may be due to the low content and very small CuS particle size. Interestingly, EDX analysis verified the coexistence of Ti, O, Cu, and S. (Fig. 3.ΙΙ.c).

**Figure 4.**
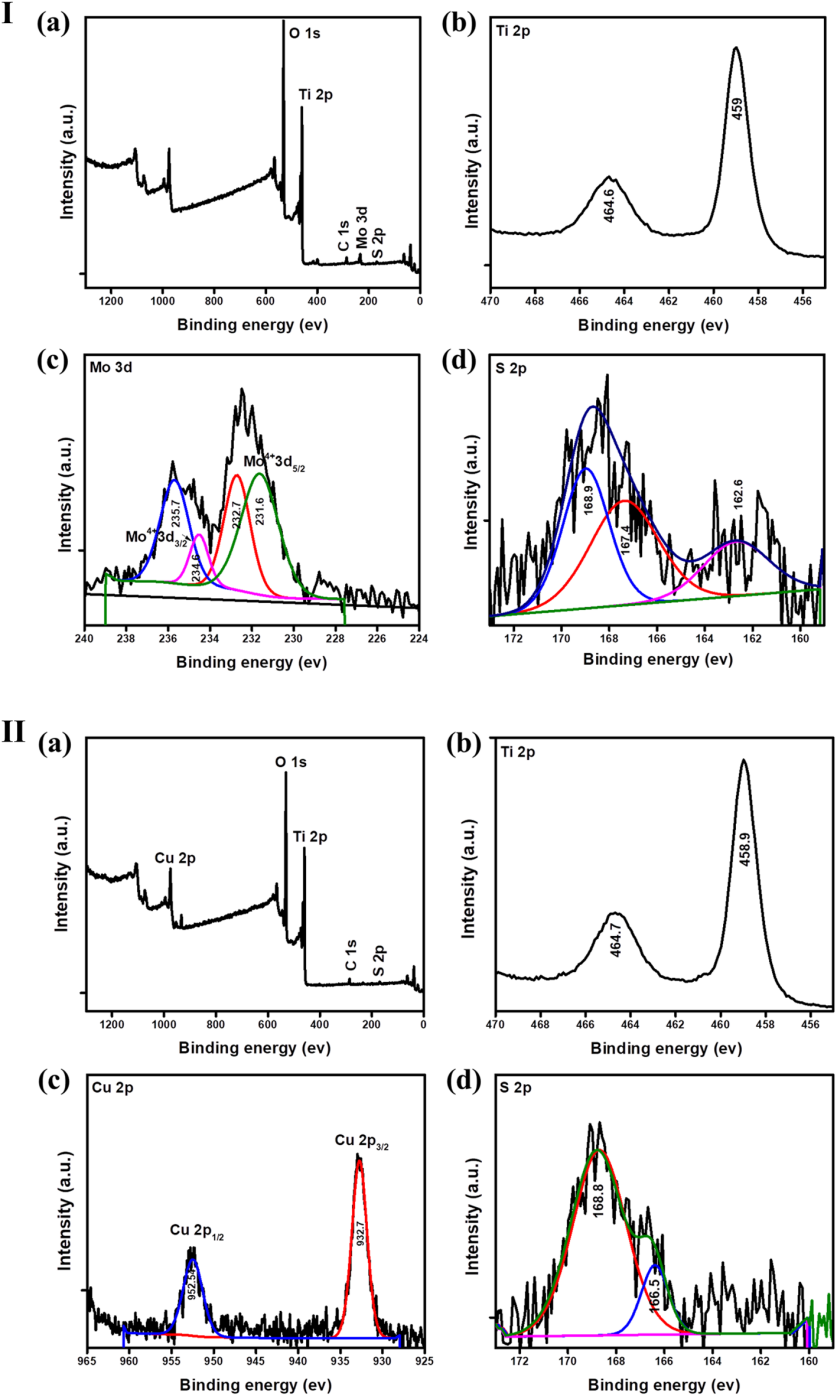
(**Ι**) XPS spectrum of the 3MT_HT nanocomposite (a) the full-scale scan, and HR-XPS spectra of (b) Ti 2p, (c), Mo 3d, and (d) S 2p. (**ΙΙ**) XPS spectrum of the 0.5CT_PP nanocomposite (a) the full-scale scan, and HR-XPS spectra of (b) Ti 2p, (c), Cu 2p, and (d) S 2p.

XPS spectra were conducted to identify the composition and chemical state of the 0.5 CT_PP and 3MT_HT nanocomposites. As observed in Fig. 4. The peak at 284.5 could be assigned to C 1s, which was used to calibrate the binding energy positions. The full survey spectrum of the as-prepared 3MT_HT (Fig. 4.Ι.a) verified the presence of C, Ti, O, Mo, and S elements. As presented in the high resolution XPS spectrum of Ti 2p, the binding energies of Ti 2p_3/2_ and Ti 2p_1/2_ were situated at 458.9 and 464.7 eV, respectively (Fig. 4.Ι.b). this indicates the existence of Ti^4+^ oxidation state in the photocatalyst. The Mo 3d HR-XPS spectrum displays the peaks at 231.4 and 234.6 eV, which may be attributed to Mo^4+^ 3d_5/2_ and Mo^4+^ 3d_3/2_ spin–orbit splitting, respectively. While the peaks positioned at 232.7 and 235.7 eV corresponded to Mo^6+^ (Fig. 4.Ι.c). Furthermore, the peak at 162.6 eV could be corresponded to S 2p_1/2_ orbitals of S^2−^, meanwhile the peaks at 167.4 and 168.4 eV possibly due to the excess of sulfur of MoS_2_ on the surface of the as-synthesized photocatalyst (Fig. 4.Ι.d)^39–42^.

The survey spectrum indicated the existence of C, Ti, O, Cu, and S elements in 0.5 CT_PP (Fig. 4.ΙΙ.a). The two peaks appeared at 458.9 and 464.7 eV were corresponded to Ti 2p_3/2_ and Ti 2p_1/2_, respectively (Fig. 4.ΙΙ.b). Furthermore, the Cu 2p high resolution XPS spectrum shows features centered at 932.7 and 952.5 eV, which corresponded to Cu^2+^ 2p_3/2_ and Cu^2+^ 2p_1/2,_ respectively (Fig. 4.ΙΙ.c)_._ The binding energy at 166.5 and 168.8 eV could be ascribed to the S 2p which indicates the existence of S^2−^ this is observed in Fig. 4.ΙΙ.d^43^.

**Figure 5:**
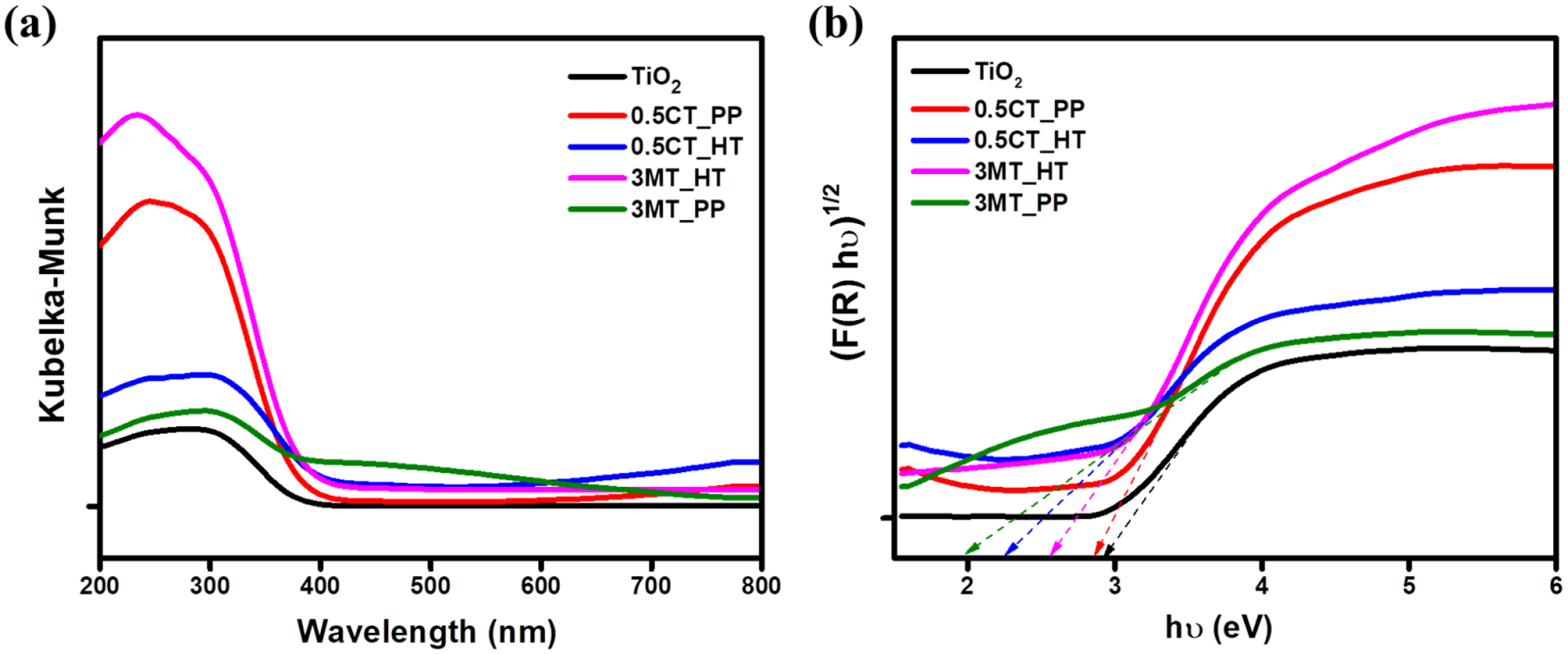
(**a**) UV − Vis absorbance spectra of TiO_2_, MoS_2_/TiO_2_ and CuS/TiO_2_ nanocomposites. (**b**) Plots of transformed KM function.

UV/Vis diffuse reflectance analysis was employed to examine the optical properties of the prepared photocatalysts. Figure 5a demonstrates the UV–Vis absorption spectra of TiO_2_ and the as prepared CuS/TiO_2_ and MoS_2_/TiO_2_ photocatalysts. It was observed that the pure P25 has absorption at around 390 nm. In the presence of CuS and MoS_2_ the absorption edges of the as-synthesized catalysts displaced to higher wavelengths which in turn increase the absorption performance in the visible light region. The photocatalysts showed a noticeable red shift of absorption with increasing the content of CuS, and MoS_2_. The UV–Vis absorption spectra different percent’s of CT_PP and MT_HT nanocomposites are shown in Fig. S3. The band gap of the as-synthesized samples was estimated using the transformed Kubelka–Munk function (Fig. 5b)^26^. The measured band gap for pure TiO_2_ was 2.92 eV. The band gaps of the 0.5CT_PP, 0.5CT_HT, 3MT_PP, and 3MT_HT catalysts were 2.83, 2.22, 1.82, and 2.55 eV, respectively. TiO_2_ modified with CuS and MoS_2_ is beneficial for electron–hole generation. Suggesting that addition of metal sulfide over TiO_2_ surface can increase the optical absorption, as metal sulfides modified TiO_2_ nanocomposites could be excited under visible light irradiation and generate additional electron–hole pairs compared to TiO_2_, which in turn enhance the photocatalytic activity.

**Figure 6:**
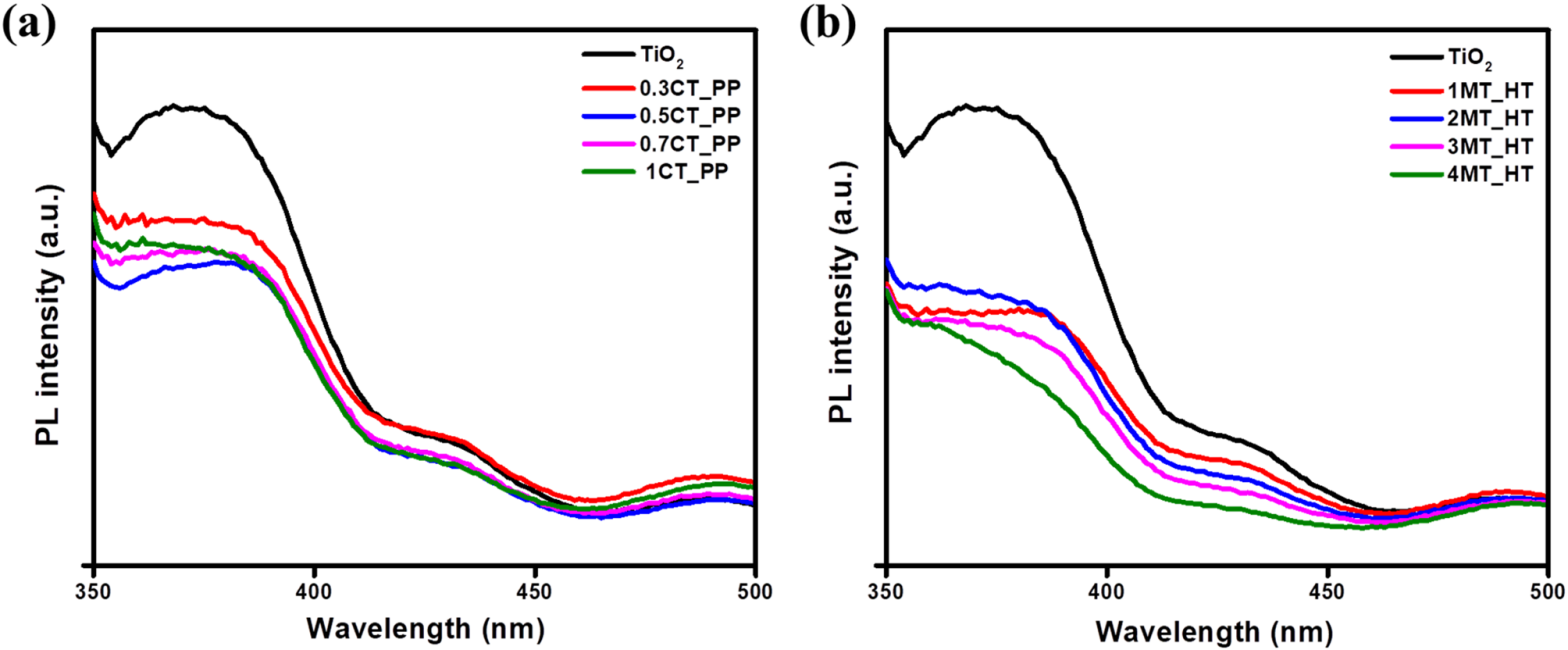
Photoluminescence spectra PL of (**a**) CT_PP, and (**b**) MT_HT compared to TiO_2_.

To further investigate the efficiency of the photogenerated electron–hole pair’s transformation and recombination process in the as-prepared samples, photoluminescence (PL) analysis was applied. Figure 6a,b show the PL spectra of TiO_2_, CT_PP, and MT_HT photocatalysts. A wide emission peak can be observed at around 370 nm. For pure TiO_2_, the PL curve intensity is greater than that of the other photocatalysts. Meanwhile, a remarkable decrease in the PL peak intensity of 0.5CT_PP and 3MT_HT was observed. In general, a lower PL intensity indicates higher separation efficiency of the photogenerated carriers. This verifies that modification of TiO_2_ with CuS or MoS_2_ can reduce the recombination rate of photoexcited carriers. Moreover, the photocatalytic activity can be improved^44^.

#### Photocatalytic H_2_ generation activity

**Figure 7:**
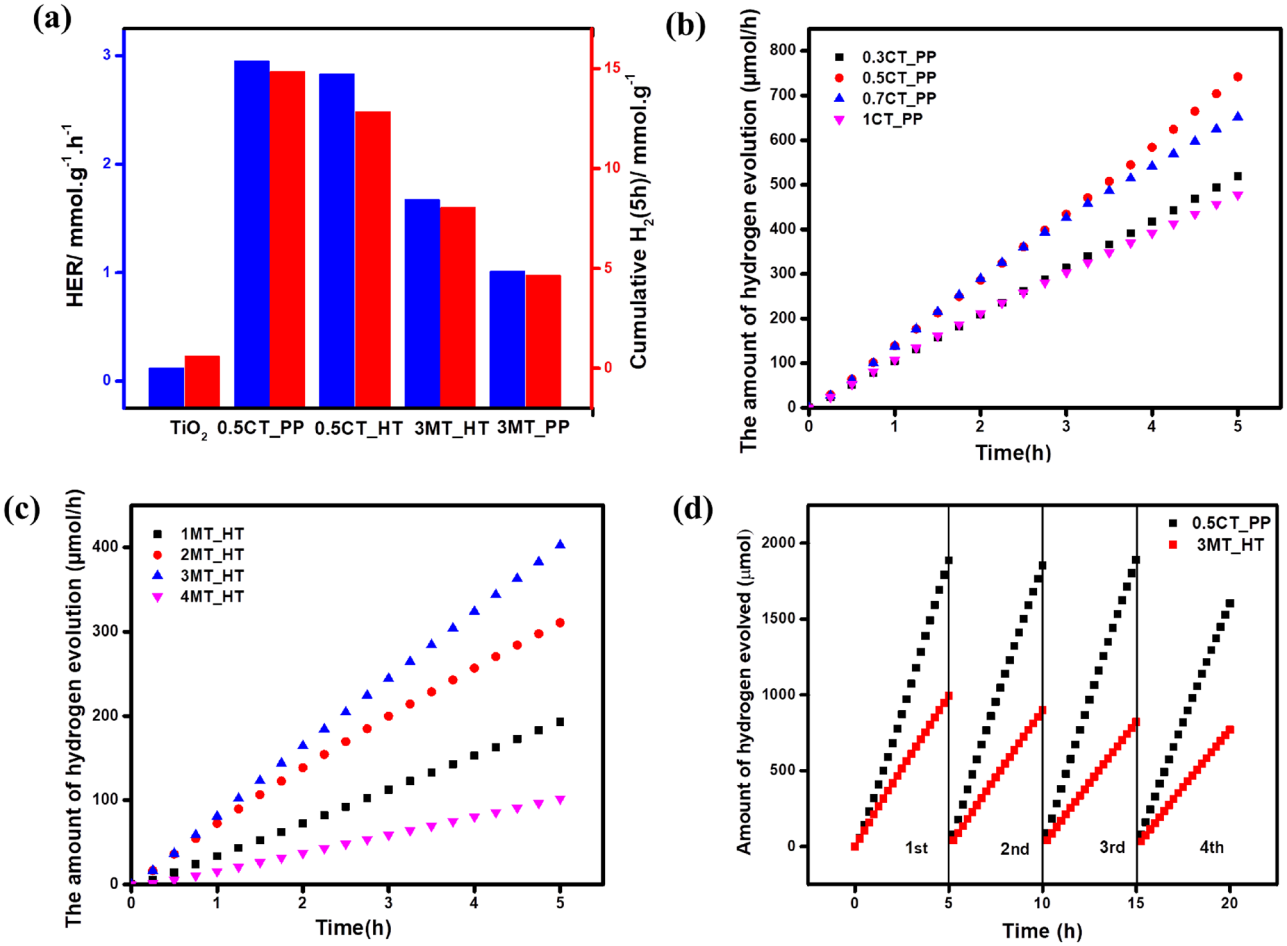
(**a**) The initial and cumulative (5 h) hydrogen evolution rate. (**b**,**c**) Time course of H_2_ evolution over of different weight percent of CuS/TiO_2_ and MoS_2_/TiO_2_, respectively, and (**d**) Recycling test of H_2_ evolution over 0.5CT_PP and 3MT_HT photocatalysts.

The photocatalytic hydrogen evolution experiments of pure TiO_2_ and the as-prepared MS_x_/TiO_2_ nanocomposites with different CuS and MoS_2_ contents were performed using an aqueous methanol solution as a scavenger agent and exposure to a UV lamb. It can be seen that pure TiO_2_ exhibited a weak HER behavior (0.086 mmol h^−1^ g^−1^) owing to its large band gap and the fast backward reaction. However, the presence of CuS or MoS_2_ led to remarkable growth in the H_2_ evolution rate. In the case of CuS/TiO_2_ catalysts, the highest activity toward H_2_ production was observed for 0.5 wt% CuS/TiO_2_ prepared by coprecipitation method with an initial hydrogen evolution rate (HER) 2.95 mmol h^−1^ g^−1^ which was thirty-five times greater than that of pure TiO_2_. Although the H_2_ production rate of 0.5 CuS/TiO_2_ prepared hydrothermally was 2.83 mmol h^−1^ g^−1^. Furthermore, the maximum H_2_ evolution rate of MoS_2_/TiO_2_ achieved 1.68 mmol h^−1^ g^−1^ when the content of MoS_2_ was 3 wt% synthesized by hydrothermal method nearly twenty times as that over pure TiO_2,_ and it was 1.013 mmol h^−1^ g^−1^ by precipitation method as presented in Fig. 7a. For comparison, different CuS and MoS_2_ mass ratios were tested over TiO_2_ under the same reaction condition (Fig. 7b,c), respectively. It is observed that increasing the amount of the metal sulfide causes a remarkable decrease in the photocatalytic performance, which is possibly due to the increased shielding of the co-catalyst on the TiO_2_ surface, which hinders the electron–hole pair transfer and decreases the exposed active sites^33,45^. The calculated AQY of photocatalytic H_2_ production over 0.5CT_PP nanocomposite was ~ 3.2% at 365 nm (Fig. S4). the stability and recyclability tests of 0.5CT_PP and 3MT_HT photocatalysts (100 mg) for H_2_ generation were performed under UV light (365 nm) for four cycles (20 h). After each cycle the reaction vessel was purged with argon gas for 1 h under dark conditions. As demonstrated in Fig. 7d, the photocatalytic activity of 0.5CT_PP and 3MT_HT remains very stable up to three cycles. Whereas, the catalytic behavior slightly decreased in the 4th cycle, which may be attributed to the consumption of the hole scavenger solution. This suggests that the prepared nanocomposite exhibit excellent stability for photocatalytic hydrogen evolution. Moreover, the XRD analysis of 0.5CT_PP and 3MT_HT before and after recycling tests was carried out. The XRD pattern preserves its original form after the photocatalytic reaction indicating the stability of the crystal structure of the prepared catalysts for long time (Fig. S5). Table 2 shows hydrogen generation rate of the prepared samples and other metal sulfide/TiO_2_ based photocatalysts.

**Table 2:** Comparison table on hydrogen generation performance of 0.5CT_PP and 3MT_HT with other transition metal sulfides/TiO_2_ based photocatalysts.

Photocatalysts	Synthesis method	Sacrificial reagent	Light source	Activity μmol h^−1^ g^−1^	Ref
MoS_2_/TiO_2_	In-situ photo-deposition	Triethanolamine	1000 W Xe arc lamp	ca. 1630	^46^
MoS_2_ nanosheets/TiO_2_ nanosheets	Hydrothermal	10 vol% methanol/H_2_O	300 W Xe lamp	2145	^47^
CuS nanoflakes/TiO_2_ nanospindles	Chemical precipitation	0.35 M Na_2_S and 0.25 M Na_2_SO_3_	300 W Xe lamp (λ > 420 nm)	1262	^29^
CuS nanoflowers/TiO_2_ NPs/Pt NPs	Hydrothermal	0.1 m Na_2_S + 0.1 m Na_2_SO_3_	400 W Xe lamp (λ > 395 nm)	746	^48^
Ag-Ag_2_S NPs/TiO_2_ NPs	In situ sulfidation of Ag	10 v% methanol/H_2_O	4 LEDs (3 W, 365 nm, 80.0 mW cm^−2^)	2382.2	^49^
CoSx quantum dots/TiO_2_	Deposition–precipitation	20 vol% ethanol	300WXe lamp	838	^50^
0.5CT_PP	Precipitation	20 vol% methanol	UV-LEDs	2950	This work
3MT_HT	Hydrothermal	20 vol% methanol	UV-LEDs	1700	This work

#### Photodegradation of MB dye

**Figure 8:**
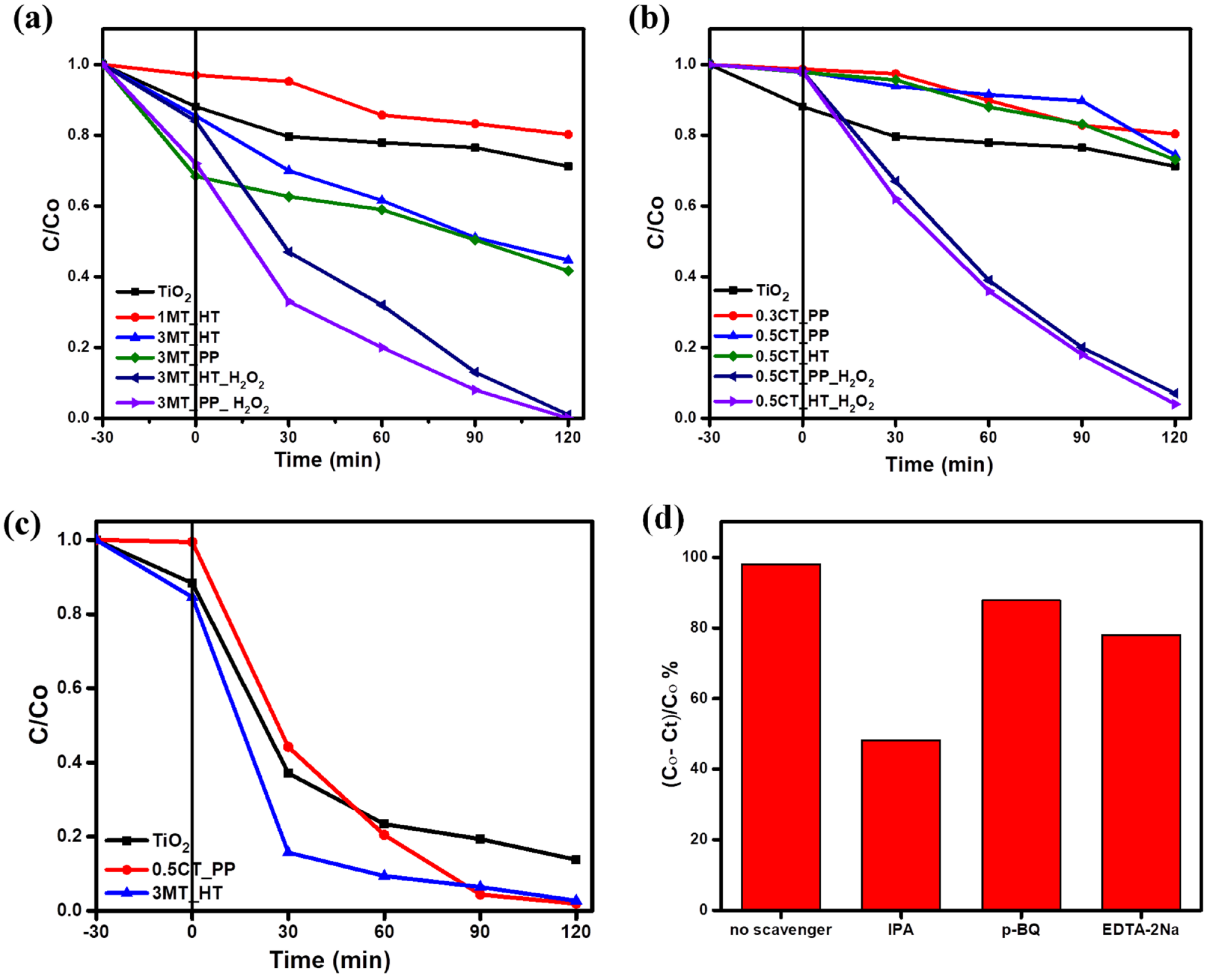
Photodegradation of MB of TiO_2_ and different percent’s of (**a**) MoS_2_/TiO_2_, (**b**) CuS/TiO_2_ with and without H_2_O_2_ under visible light. (**c**) Photodegradation behavior of MB of TiO_2_, 0.5CT_PP, and 3MT_HT under UV–Vis light. (**d**) Trapping experiments of active species over 3MT_HT (pH 6.5, 50 mg of catalyst, 50 ppm MB).

The photocatalytic performance of CuS/TiO_2_ and MoS_2_/TiO_2_ photocatalysts can also be assessed by the photodegradation of MB solutions under both visible and UV irradiation light using a 450 W medium-pressure mercury lamp with a < 420 nm UV cut-off filter for two hours. First, the synthesized catalysts were exposed to a 50 mg/L MB dye solution for 30 min in the dark conditions to reach adsorption–desorption equilibrium. MoS_2_/TiO_2_ nanocomposites had superior efficiency toward MB degradation under visible light (Fig. 8a). After 120 min visible light irradiation, 3MT_HT exhibits a significant photocatalytic performance compared to P25. The appropriate amount of MoS_2_ has a great influence on enhancing the catalytic activity of TiO_2_. After exposure to visible light, the degradation efficiency of MB dye over P25 was enhanced from 28.8 to 58.33% over 3MT_PP. The band gap of TiO_2_ P25 shifted from the UV region (2.93 eV) to the visible region (1.82 eV) on 3MT_PP, as shown in Table 1. A further increase in the photocatalytic activity has observed by the injection of 5 mL H_2_O_2_ in the suspension leading to an efficiency of 100% and 99% over 3MT_PP and 3MT_HT after 120 min visible light irradiation (Fig. 8a), respectively. It has been found that CuS/TiO_2_ nanocomposites had minuscule adsorption ability toward the MB solution compared to P25 (Fig. 8b). For comparison, the photodegradation percentages of MB solution under the visible light irradiation were 19%, 18%, 24.5%, and 25.3%, for p25, 0.3CT_PP, 0.5CT_PP, and 0.5CT_HT, respectively, suggesting that the formation of a heterostructure between CuS and TiO_2_ can enhance the photocatalytic performance, Among those, 0.5CT_HT shows the highest photocatalytic efficiency because its band gap becomes 2.22 eV (Table. 1). Furthermore, the addition of H_2_O_2_ increased the photodegradation , and the removal efficiency reached 93%, and 96% for 0.5CT_PP and 0.5CT_HT, respectively^51^. On the other hand, the photodegradation of MB aqueous solution under the illumination of UV–Vis light is illustrated in Fig. 8c. The results indicate that the photocatalytic activity was improved at 0.5CT_PP and 3MT_HT which rose to nearly 98%, indicating that CuS and MoS_2_ can form a heterojunction with TiO_2_, which can, in turn, enhance the photocatalytic activity. The photodegradation of MB by MS/TiO_2_ based photocatalysts are summarized in Table 3.

**Table 3:** Comparison table on photodegradation of MB of the as-prepared photocatalysts with metal sulfides modified TiO_2_.

Photocatalysts	Synthesis method	Pollutant content/(mg L^−1^)	Light Source	% of degradation	Ref
MoS_2_/TiO_2_	Hydrothermal method	5 mg/L	400 W Xe lamp visible light	99.33	^52^
Cu_x_S/TiO_2_	In situ synthesis	10 mg/L	Visible	95	^53^
CdS/TiO_2_	SILAR method	12 mg/L	160 W Hg lamp	93.8	^54^
PbS/GR/TiO_2_	Sol–gel method	1 × 10^-4^ M	Visible	41	^55^
CuS/TiO_2_ nanofiber	Electrospinning and hydrothermal processes	10 mg/L	Visible	79.09	^56^
0.5CT_HT	Hydrothermal	50 mg/L MB + H_2_O_2_	450 W Hg lamp visible light	96	This work
3MT_PP	Precipitation	50 mg/L MB + H_2_O_2_	450 W Hg lamp visible light	100	This work

To investigate the contribution of active species during the photocatalytic degradation of MB, elemental trapping experiments have been performed in which, isopropanol (IPA), p-benzoquinone (BQ) and ethylene diamine tetraacetic acid disodium (EDTA-2Na) were used as scavengers to quench the free radical hydroxide ⋅OH, superoxide radical ⋅O_2_^‒^ and photogenerated holes h^+^, respectively. As shown in Fig. 8d, the photodegradation of MB over 3MT_HT without any scavenger reached 98% under UV–Vis irradiation for 2 h. The addition of IPA decreased the activity to 48.2%, while the degradation efficiency quenched to 87.8 and 78% when using p-BQ and EDTA-2Na, respectively. These results indicate that ⋅OH radical is the major active species for the dye degradation reaction. However, h^+^ and ⋅O_2_^‒^ have a minor effect on the photocatalytic process. The effect of catalyst amount and the initial pH of the solution are presented in Fig. S6. The pH of the solution was adjusted using diluted HCl solution and diluted NaOH solution.

### Photoelectrochemical measurements

**Figure 9:**
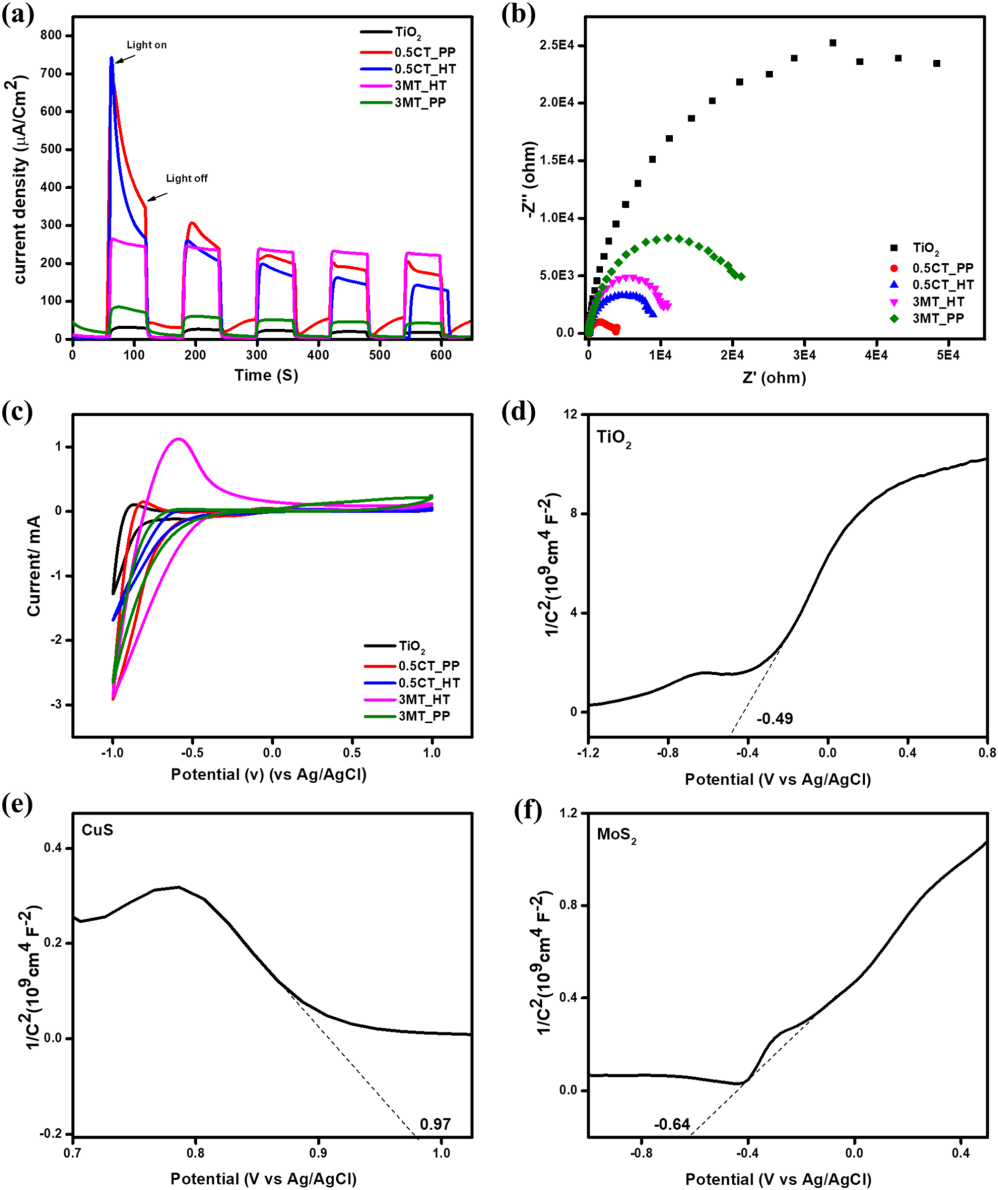
(**a**) Transient photocurrent response (i−t); (**b**) EIS Nyquist plots, (**c**) cyclic voltammetry curves of TiO_2_, 0.5CT_PP, 0.5CT_HT, 3MT_HT and 3MT_PP photocatalysts, Mott–Schottky plots of pure (**d**) TiO_2_, (**e**) CuS, and (**f**) MoS_2_.

Electrochemical measurements using EIS, CAM, and CV were carried out to validate the improved separation efficiency of the electron–hole pair in metal sulfides modified TiO_2_ nanocomposite^57^. The transient photocurrent responses of the as-prepared electrodes were measured under UV light irradiation at a bias potential of 0.6 V (vs. Ag/AgCl). In a 0.1 M Na_2_SO_4_ electrolyte solution. The photocurrent response increased immediately as the light was turned on and decreased to zero after turning off the light. The photocurrents of 0.5CT_PP, 0.5CT_HT, 3MT_HT and 3MT_PP were better than pristine TiO_2_, as shown in Fig. 9a. The 0.5CT_PP and 3MT_HT nanocomposites were 22 and 12 times higher than that of pure TiO_2_, respectively. Hence, heterojunction formation between metal sulfides and TiO_2_ leads to enhanced charge separation. Moreover, the photocurrent response exhibited reproducibility, representing the high stability of the as-prepared photocatalysts. Figure 9b displays the EIS Nyquist plots of TiO_2_, 0.5CT_PP, 0.5CT_HT, 3MT_HT, and 3MT_PP under UV light irradiation. EIS was used to study the charge separation efficiency. The semi-circle refers to the charge transfer resistance across the interface. Impedance fitting revealed a significantly reduced semi-circle of 0.5CT_PP, indicating that 0.5CT_PP has a significantly improved charge transfer efficiency, enhanced conductivity, and superior separation of photogenerated charges, resulting in an improved photocatalytic performance of the as-synthesized photocatalysts than that of pure TiO_2_. Furthermore, CV was performed to estimate the catalytic performance and the reduction sites of the prepared metal sulfide/TiO_2_ catalysts. The estimated reduction currents for TiO_2_, 0.5CT_PP, 0.5CT_HT, 3MT_HT and 3MT_PP were 1.30, 2.94, 1.71, 2.90, and 2.66 mA cm^−2^, respectively (Fig. 9c), indicating that the cathodic current has been improved.

Mott–Schottky (MS) analysis was used to investigate the conductivity and flat band potentials (E_FB_) of pure CuS, MoS_2_, and TiO_2_^58^. TiO_2_ and MoS_2_ are n-type semiconductors while CuS is a p-type semiconductor (Fig. 9d–f). The measured (E_FB_) for TiO_2_, MoS_2_ and CuS were − 0.49, − 0.64 and 0.97 V *vs.* Ag/AgCl, respectively. These values can be transformed to the normal hydrogen electrode (NHE) scale using the following equation: E_NHE_ = E_Ag/AgCl_ + 0.197.

### Photocatalytic mechanism

**Figure 10:**
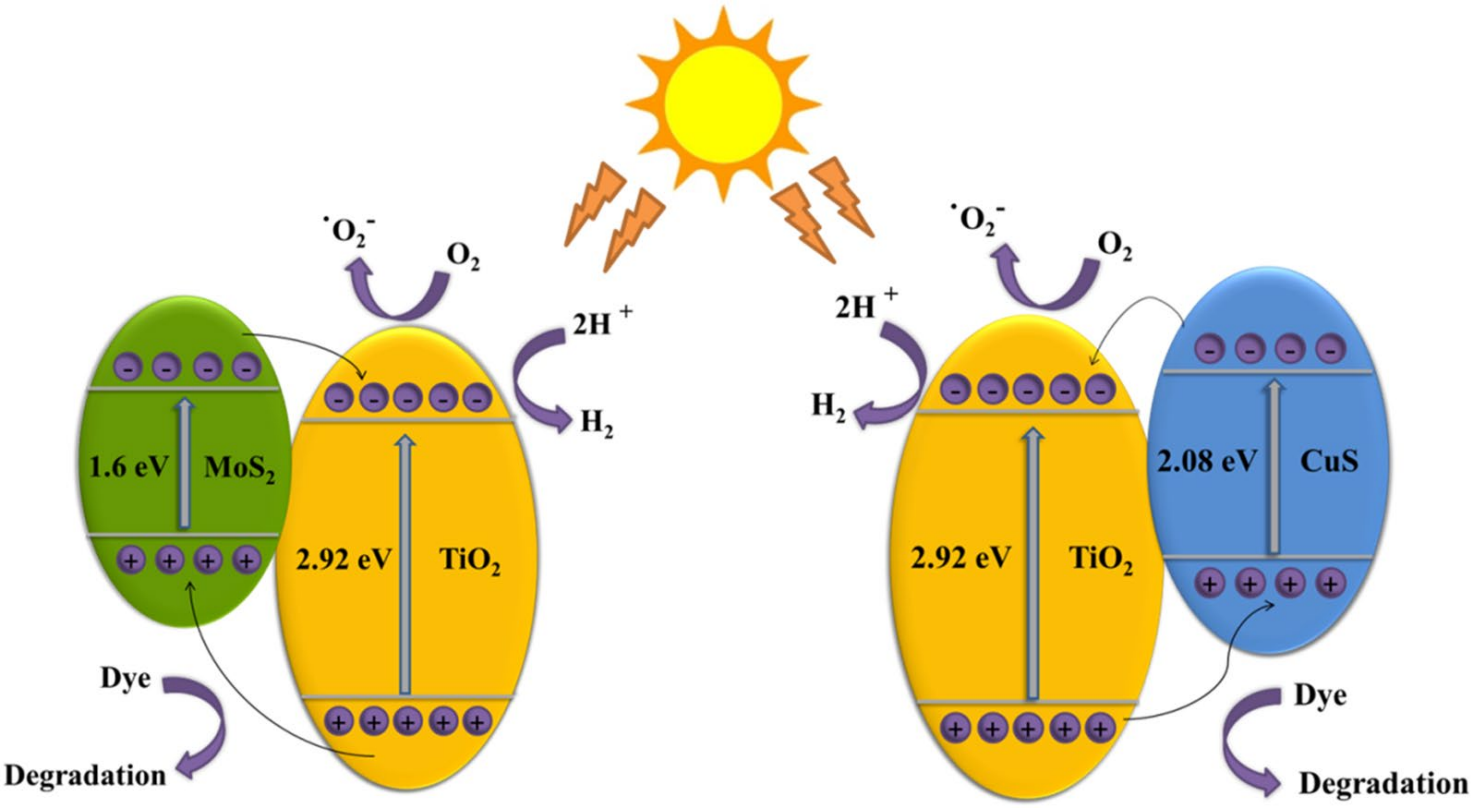
Scheme illustrating the photocatalytic mechanism of CuS/TiO_2_ and MoS_2_/TiO_2_.

In the light of the previous discussion, the possible mechanism for H_2_ generation and MB degradation over the as-prepared metal sulfides doped TiO_2_ photocatalysts is shown in Fig. 10. Under light illumination, TiO_2_ modified by CuS or MoS_2_ was excited, leading to the generation of charge carriers. The photoexcited electrons on the CB of CuS or MoS_2_ could transfer directly to the CB of the TiO_2_, because the CB edge potentials of CuS (− 0.71) and MoS_2_ (− 0.64) are more negative than TiO_2_ (− 0.49). Meanwhile, the VB level of TiO_2_ (2.51) is more positive than that of CuS (1.37) and MoS_2_ (0.96), thus the photoexcited holes migrate in the reverse direction of electrons. The band gaps of CuS and MoS_2_ are displayed in Fig. S7a,b. Based on the band alignment data, the heterojunction between the metal sulfide and TiO_2_ follow type II. This could be further verified via elemental trapping experiments in the MB degradation. It was found that the primary active species is the free radical hydroxide ⋅OH for the photodegradation reaction. Whereas, superoxide radical ⋅O_2_^‒^ was minor active species and didn’t contribute sufficiently in the photodegradation of MB^59,60^. On the other hand, under UV irradiation, photogenerated electrons produced by metal sulfides could transfer to the surface of TiO_2_ resulting in the reduction of 2H^+^ to H_2_, while the photogenerated holes react with methanol. The photodegradation of MB under UV–Vis and only Vis light irradiation involves the capture of the photogenerated electrons accumulated on the CB of TiO_2_ by an O_2_ molecule to produce ⋅O_2_^−^. In the presence of H_2_O_2_, it can interact to generate ⋅OH to degrade MB. Meanwhile, the photogenerated holes accumulated in the VB of CuS and MoS_2_ react with OH^−^ and adsorbed organics to produce ⋅OH. Bare wide band gap TiO_2_ has poor photocatalytic activity due to the high recombination rate of electron–hole pairs and crystal defects. Loading of metal sulfide on the surface of TiO_2_ could improve the interfacial charge transfer, suppress charge recombination, and inhibit the backward reaction. This in turn causes a substantial increase in the catalytic performance toward both H_2_ evolution and dye degradation^1,61^.

## Conclusion

In summary, CuS/TiO_2_ and MoS_2_/TiO_2_ photocatalysts have been successfully synthesized by facile coprecipitation and hydrothermal method with different weight percent’s, which exhibited a superior photocatalytic activity toward H_2_ generation and MB degradation compared to TiO_2_. Various techniques were used to characterize the as-prepared catalysts. The optimal CuS loading content to TiO_2_ was 0.5wt% prepared by coprecipitation method with a hydrogen evolution rate of 2.95 mmol h^−1^ g^−1^ and the degradation efficiency of MB was 93% in the presence of H_2_O_2_ under visible light. Meanwhile, the maximum photocatalytic hydrogen production rate of TiO_2_ modified with MoS_2_ was achieved by 3MT_HT (1.7 mmol h^−1^ g^−1^) and the photodegradation of MB was 99%. To further investigate the superior charge carrier transfer and the photocatalytic activity of 0.5CT_PP and 3MT_HT, photoelectrochemical measurements (PEC) were undertaken. Under light irradiation (365 nm), from EIS study, the Nyquist plots of the samples show that 0.5CT_PP had the smallest semicircle arc indicating the highest charge carrier separation and excellent charge transport characteristics. This plays a crucial role in enhancement its photocatalytic activity for hydrogen generation. Also, the photocatalytic activity can be evaluated using cyclic voltammetry (CV) measurements, where 0.5CT_PP had the highest cathodic current which indicates that TiO_2_ loaded by 0.5 wt% CuS prepared via co-precipitation method has the greatest photocatalytic H_2_ performance. In conclusion, coupling of metal sulfides with TiO_2_ enhances the separation of the photoinduced electrons and holes, which in turn improve the photocatalytic performance.

## Supplementary Information

Supplementary Figures.

## Data Availability

All data generated or analyzed during this study are included in this published article (and its supplementary information files).
