# Supplementary material for: Metal chalcogenides (CuS or MoS2)-modified TiO2 as highly efficient bifunctional photocatalyst nanocomposites for green H2 generation and dye degradation
Source: Sci Rep. 2023 May 17;13:7994. doi: 10.1038/s41598-023-34743-2 (PMC10192425; doi:10.1038/s41598-023-34743-2)
Supplement: Supplementary file 1 — Supplementary Figures. [file 41598_2023_34743_MOESM1_ESM.docx]

**Supporting Information for**

**Metal chalcogenides (CuS or MoS_2_)-modified TiO_2_ as highly efficient bifunctional photocatalyst nanocomposites for green H_2_ generation and dye degradation**

**Reem A. El-Gendy*^1,2^, Haitham M. El-Bery*^1,2^, Mostafa Farrag^3^, Dina M Fouad^3^**

^1^ Advanced Multifunctional Materials Laboratory, Chemistry Department, Faculty of Science, Assiut University, Assiut 71515, Egypt.

^2^ Basics Science Department, School of Biotechnology, Badr University in Assiut, Assiut 2014101, Egypt.

^3^ Department of Chemistry, Faculty of Science, Assiut University, Assiut, 71515, Egypt

***Corresponding Author Email:** [**reem.abdellah@bua.edu.eg**](mailto:reem.abdellah@bua.edu.eg)

***Corresponding Author Email:** [**Haitham.El-Bery@aun.edu.eg**](mailto:Haitham.El-Bery@aun.edu.eg)


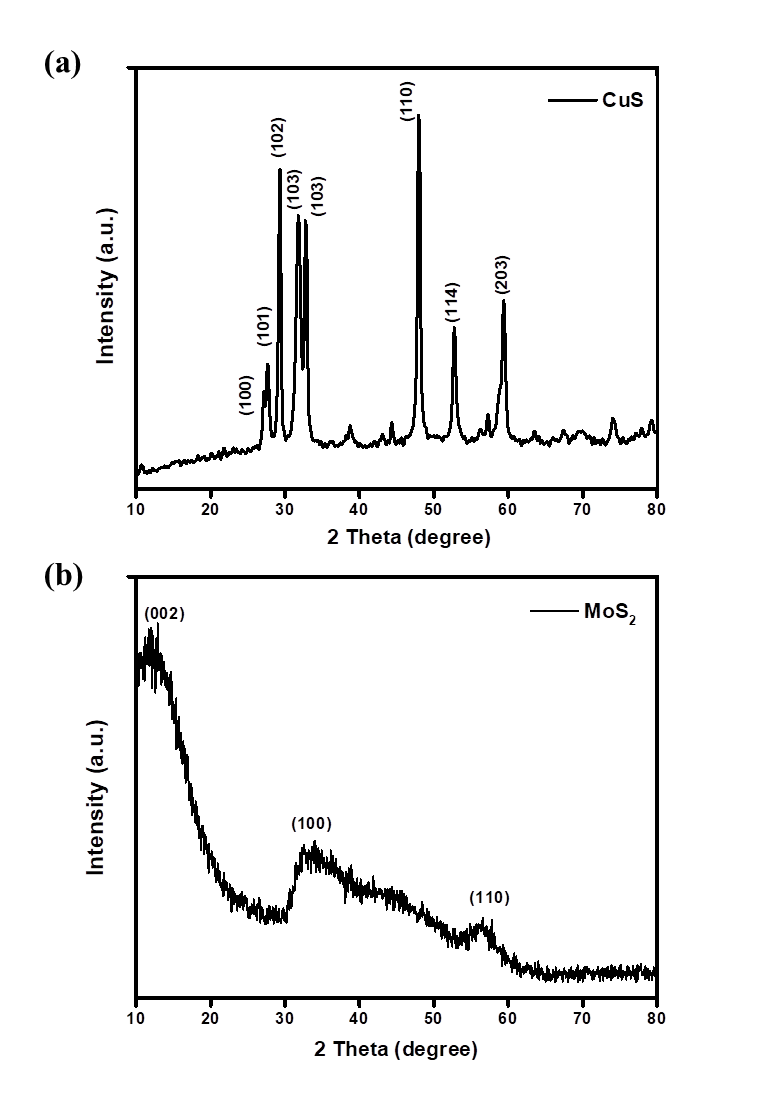


**Fig. S.1. XRD patterns of (a) CuS, and (b) MoS_2_**


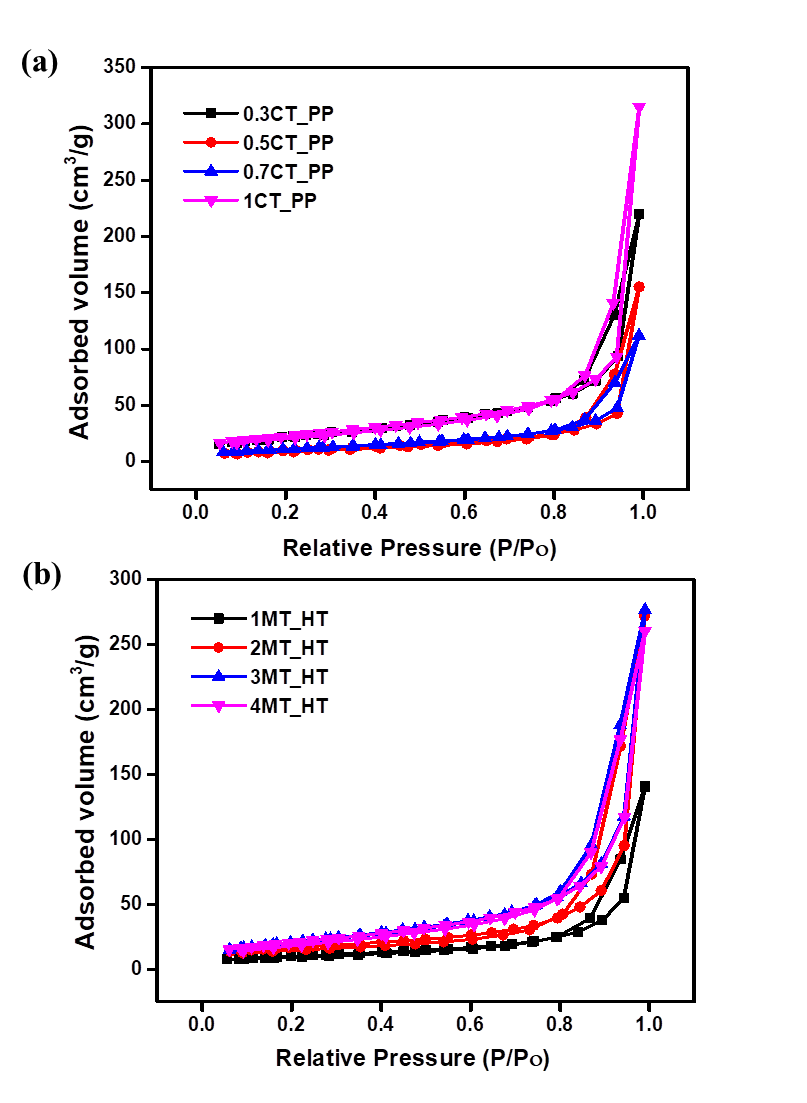


**Fig. S.2. N_2_ adsorption/desorption isotherm of different percent’s of (a) CuS, and (b) MoS_2_**


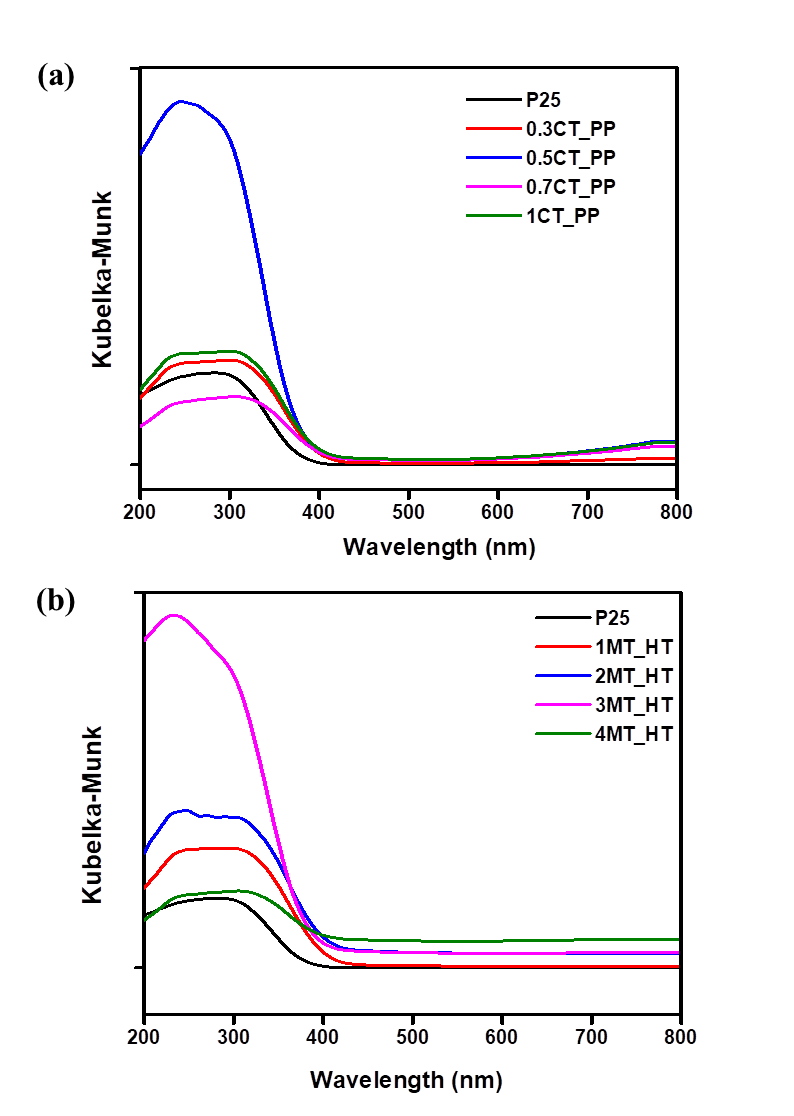


**Fig. S.3. UV−Vis absorbance spectra of different percent’s of (a) CuS, and (b) MoS_2_**


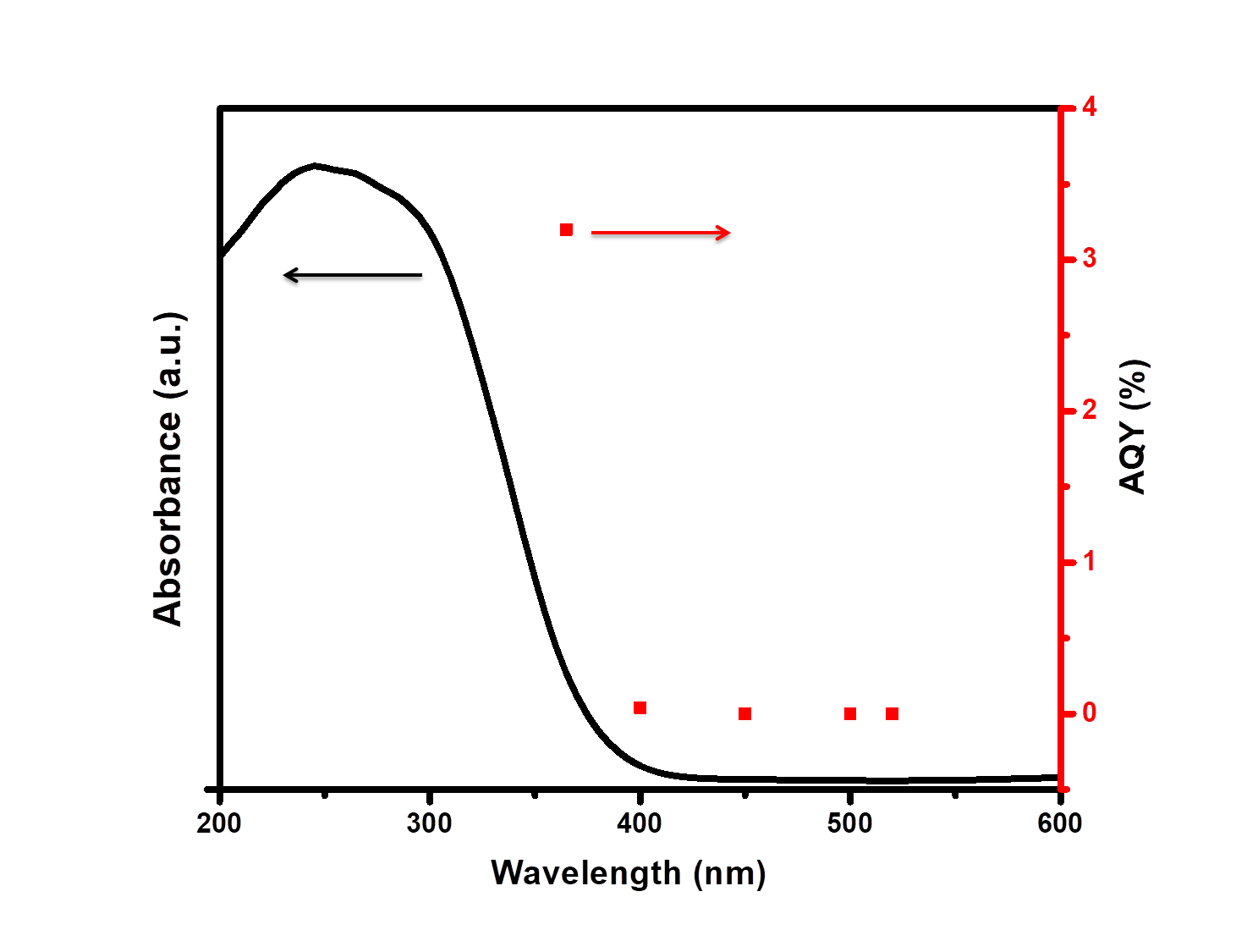


**Fig. S.4. Wavelength-dependent AQY of H_2_ evolution over 0.5CT_PP photocatalyst.**

**Fig. S.5. XRD pattern of (a, c) 0.5CT_PP and 3MT_HT before recycling, respectively, (b, d) 0.5CT_PP and 3MT_HT after recycling, respectively.**


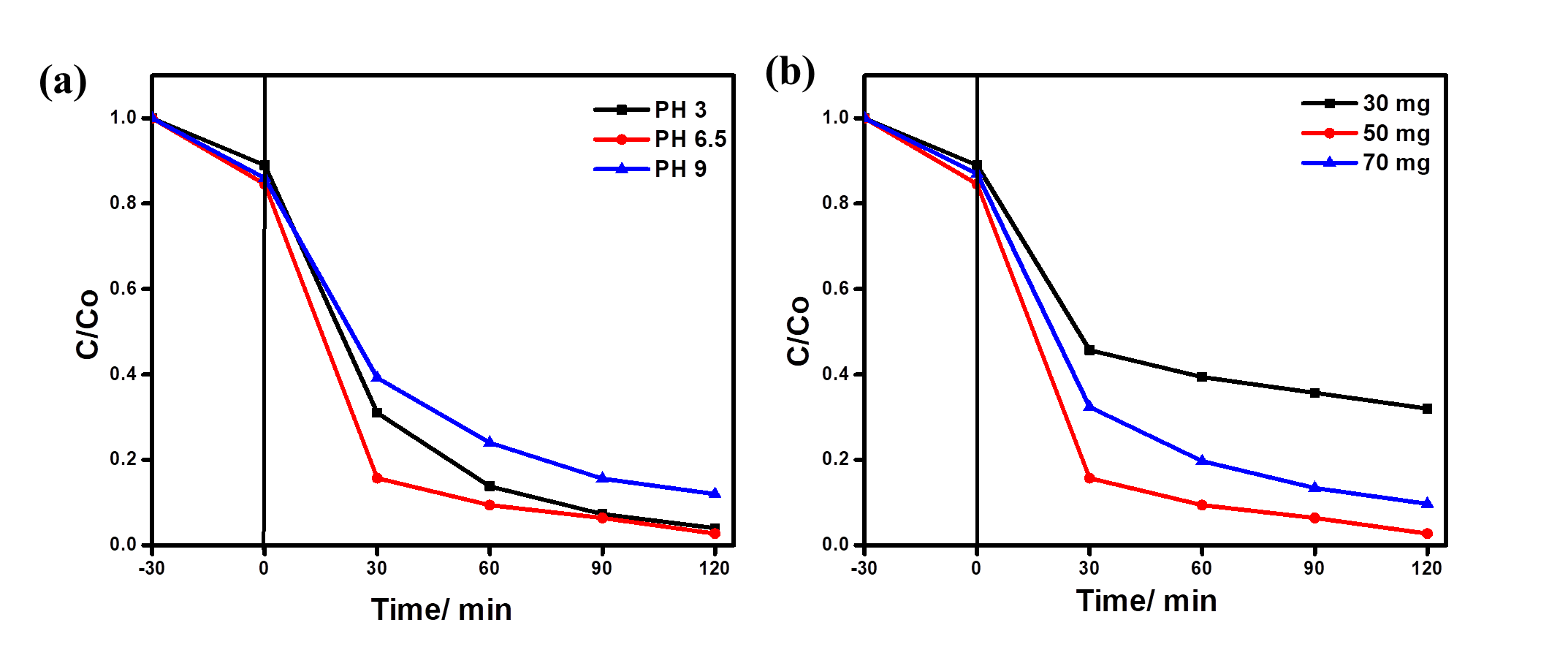


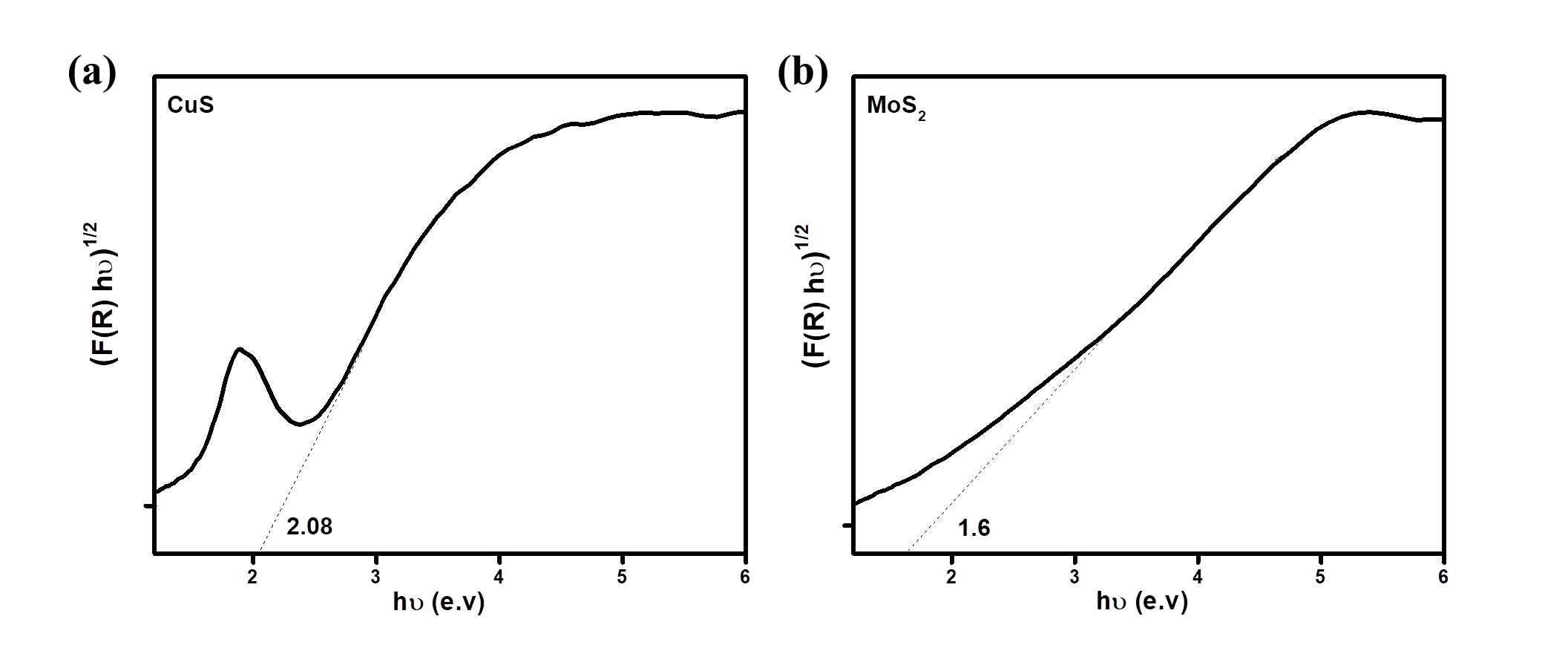
**Fig. S.6. The effect of (a) initial PH, (b) various amount of 3MT_HT toward MB solution.**

**Fig. S.7. Plots of transformed KM function of (a) CuS, and (b) MoS_2_**


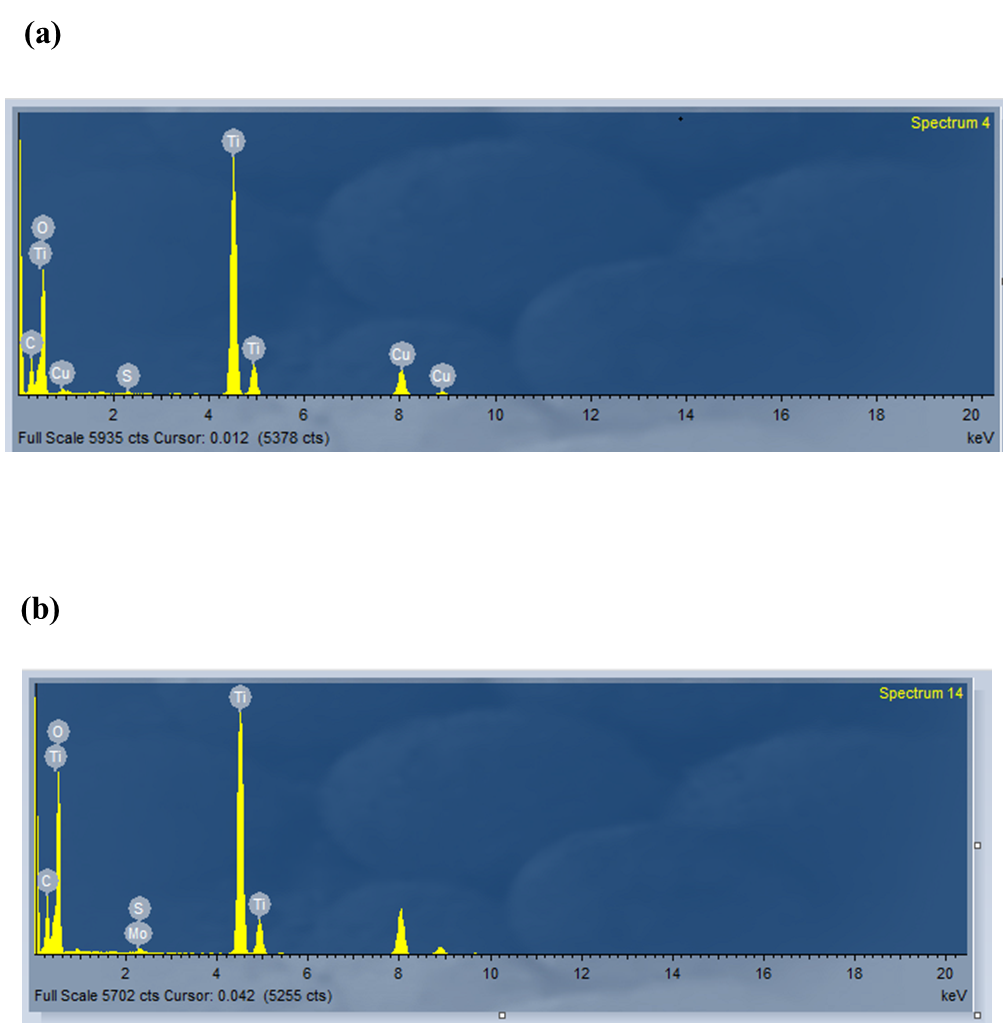


**Fig. S.8. EDX spectrum of (a) 0.5CT_PP, and (b) 3MT_HT.**
